# Inflammatory Markers: C-Reactive Protein, Erythrocyte Sedimentation Rate, and Leukocyte Count in Vitamin D Deficient Patients with and without Chronic Kidney Disease

**DOI:** 10.1155/2013/802165

**Published:** 2013-06-26

**Authors:** Ibrahim Yildirim, Ender Hur, Furuzan Kokturk

**Affiliations:** ^1^Division of Nephrology, Department of Internal Medicine, University of Bulent Ecevit, 67600 Zonguldak, Turkey; ^2^Department of Biostatistics, University of Bulent Ecevit, 67600 Zonguldak, Turkey

## Abstract

Although some studies revealed a positive relationship between vitamin D_3_ deficiency and inflammatory markers, there have been also many studies that failed to find this relationship. The aim of this large scaled study is to determine the association between the level of plasma 25 hydroxy vitamin D_3_ [25-(OH) D_3_] and inflammatory markers in the general population without chronic kidney disease (CKD) and in patients with CKD. Participants with simultaneously measured inflammatory markers and 25-(OH) D_3_ levels were retrospectively analyzed (*n* = 1897). The incidence of all-cause inflammation infection, hospitalization, chronic renal failure, and vitamin B12 deficiency was evaluated. The medians of serum creatinine levels in subjects without renal failure were lower in 25-(OH) D_3_ deficient group. Patients with CKD were more likely to have vitamin D_3_ deficiency compared with normal GFR. 25-(OH) D_3_ levels were associated with a greater incidence of all-cause hospitalization, hypoalbuminemia, and vitamin B12 deficiency. However, there was no relationship between inflammatory markers and vitamin D_3_ levels. In 25-(OH) D_3_ deficient patients, inflammatory markers can be related to other inflammatory and infectious status such as malnutrition and cachexia. We believed that there must be a relationship between vitamin deficiency and inflammatory markers due to other causes than low 25-(OH) D_3_ status.

## 1. Introduction

The deficiency of vitamin D_3_ is commonly associated with chronic kidney disease (CKD), and the prevalence of this hypovitaminosis increases as kidney function declines [1, 2]. Several factors, such as aging, loss of appetite, and other factors affecting cutaneous synthesis, such as low sun exposure and skin pigmentations [3], have consistently been associated with low 25-hydroxyvitamin D [25-(OH) D_3_] levels in the general population. Therefore, it is common in the elderly, malnourished individuals, and some societies [4].

Even though there is growing evidence to suggest that vitamin D_3_ status is associated with the development and progression of cardiovascular disease [5, 6], diabetes [7], and immune system disorders [8], there is limited information about the association of 25-(OH) D_3_ deficiency and inflammation in the general population without CKD and in patients with CKD.

Studies examining the association between low 25-(OH) D_3_ levels and inflammation infection are still popular. There are studies suggesting a relationship between a lack of vitamin D_3_ and morbidity and also mortality as well [9–12]. The results of these studies were contradictory and confusing. Randomized controlled trials of vitamin D_3_ supplementation have shown incompatible results, with some trials suggesting a decrease [13, 14] and other studies concluding no effect on inflammatory biomarkers [15].

The potential relationship between the deficiency of vitamin D_3_ and infection-inflammation remains poorly understood. Therefore, the aim of present study is to examine the association between the level of plasma 25-(OH) D_3_ and inflammatory markers in the general population without chronic kidney disease and in patients with CKD.

## 2. Methods

### 2.1. Study Population

Present study was conducted between January 1, 2008 and April 25, 2012 in Bulent Ecevit University Hospital and 1897 patients with 25-(OH) D_3_ levels and inflammatory markers measured simultaneously were included. Patients whose age under 18 years, patients with primary hyperparathyroidism and hypoparathyroidism, were excluded. The study participants' age, gender, and hospitalization data were recorded. The clinical and laboratory data are shown in Tables 1 and 2. The relationship between 25-(OH) D_3_ levels and serum creatinine, parathormone (PTH), sensitive C-reactive protein (CRP), erythrocyte sedimentation rate (ESR), leucocyte count, platelet count, and hemoglobin concentrations were evaluated as retrospectively in this study population.

**Figure 1:**
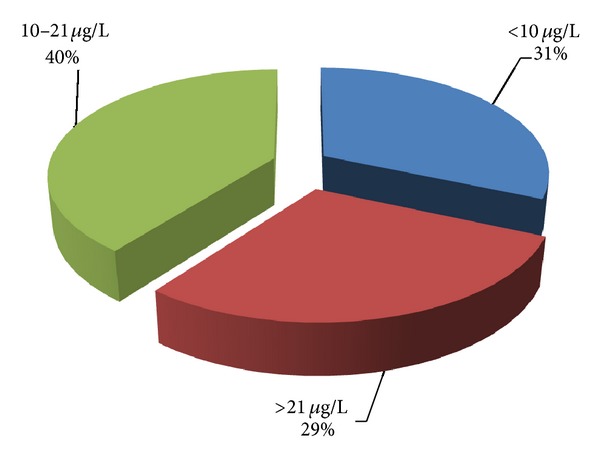
Distribution of 25-(OH) vitamin D_3_ levels in study population.

Serum 25-(OH) D_3_ levels vary depending on season; we categorized patients into two groups according to serum 25-(OH) D_3_ levels. Group 1 was composed of vitamin D_3_ deficient (<10 *μ*g/L) population, and Group 2 was composed of vitamin D_3_ normal group (>21 *μ*g/L). Patients with vitamin D_3_ levels between 10 *μ*g/L and 20 *μ*g/L were excluded from the analysis in order to avoid the effects of seasonal changes. So this intermediate group was not used in this study (Figure 1).

Patients with known levels of CRP were grouped categorically as normal (CRP < 6 mg/L; there was no inflammation or infection) and as abnormal (CRP > 30 mg/L; there was important inflammatory or infectious status). Likewise, to examine the relationship between renal failure and 25-(OH) D_3_ levels, participants were divided into categorical groups: the patients with and without renal failure. CKD was defined according to serum creatinine levels. Study cases with serum creatinine levels above 1.3 mg/dL for more than 3 months were considered as patients with CKD.

Moreover, the participants in this study were also divided into two groups: ambulatory patients and hospitalized patients.

Finally, vitamin B12 levels were measured in vitamin D_3_ deficient and vitamin D_3_ normal group, and then these two groups were divided into subgroups of their own.

Primary endpoints are as follows:

1. determining the 25-(OH) D_3_ level in the general population and in patients with CKD;
2. comparing the clinical and laboratory data regarding inflammation with levels of 25-(OH) D_3_;
3. evaluation of whether low and normal 25-(OH) D_3_ levels and inflammation could explain this potential association;

### 2.2. Biochemical Analysis

25-Hydroxyvitamin D_3_ levels were measured by high performance liquid chromatographic analysis performed with using a Zivak HPLC system (Gebze, Turkey) using a commercial 25-OH vitamin D_3_ kit (Recipe, Munich, Germany). The reference values were 10–50 *μ*g/L for winter, 20–120 *μ*g/L for summer seasons. A deficiency in 25-(OH) D_3_ level was considered as below 10 *μ*g/L.

Serum vitamin B12 and plasma PTH levels were measured with chemiluminescence method by Immulite 2000 (Diagnostic Products Corp., LA, USA).

PTH was measured by chemiluminescence with reference values of 16–87 *μ*g/L.

C-reactive protein was assayed with Dade Behring BN ProSpec System using a nephelometric method.

Serum albumin levels and ESR were measured by routine laboratory methods.

Leukocyte count, platelet count, and hemoglobin concentrations were measured by Beckman Coulter LH 780 hematology analyzer.

### 2.3. Statistical Analysis

Statistical analyses were performed by SPSS 18.0 software (SPSS Inc., Chicago, IL, USA). Distribution of data was determined by Kolmogorov-Smirnov test. Continuous variables were expressed as median (minimum-maximum) and categorical variables as frequency and percent. Continuous variables were compared with the Mann-Whitney *U* test and categorical variables were compared using Pearson's Chi-square test. Linear relation between two continuous variables was evaluated by Spearman correlation analysis. *P* value of less than 0.05 was considered statistically significant for all tests.

## 3. Results

A total of 1897 subjects were included in this retrospective study. Patients that measured 25-(OH) D_3_ levels under 10 *μ*g/l were 598 (31.5%), the number of those between 10 and 21 *μ*g/L was 751 (39.5%), and the number of those over 21 *μ*g/L was 550 (28.9%), respectively, in the study group (Figure 1).

The difference between male and female in 25-(OH) D_3_ levels was statistically significant (*P* < 0.001), and 25-(OH) D_3_ levels were significantly lower in female [16.1 ± 12.8 (3–121)] than in male [19.2 ± 12.9 (3–201)]. For this reason, male and female patients were divided into groups according to the presence of renal failure. The results are summarized in Tables 3 and 4.

There was no significant correlation between age and vitamin D_3_ deficiency in our study population. There were lower serum albumin levels in patients with vitamin D_3_ deficiency, but this was not statistically significant (Table 3). Median serum creatinine levels were less in patients with vitamin D_3_ deficiency without renal failure than in participants with normal vitamin D_3_ levels without renal failure (Table 4). 

Serum albumin, CRP, ESR, and WBC levels had no significant relationship in groups that vitamin D_3_ deficiency and vitamin D_3_ normal in male and female patients without renal failure (Tables 1–4). There was no difference in the levels of albumin, CRP, ESR, and WBC in women with renal insufficiency, but there was significant difference between levels of serum albumin and ESR in male patients.

The inflammatory status measured by CRP showed no difference with respect to the 25-(OH) D_3_ (*P* = 0.318).

In CRP variable that was categorized as <6 mg/L and ≥6 mg/L, there was no significant difference between CRP categories and 25-(OH) D_3_ levels (*P* = 0.728). The results are summarized in Table 5.

In CRP variable that was categorized as <6 mg/L and ≥30 mg/L, there was no significant difference between these CRP categories and 25-(OH) D_3_ levels (*P* = 0.635) (Table 6).

Then, all participants were included in the study, both 25-(OH) D_3_ and CRP variables taken as a numerical, and correlation analysis was performed. There was no correlation between the two groups (*r* = −0.03, *P* = 0.335).

The difference between men and women CRP levels was statistically significant (*P* = 0.013).

CRP and vitamin D_3_ levels in men and women were different; therefore, similar analyses were repeated in men and women groups. Median CRP in vitamin D_3_ deficient group and vitamin D_3_ normal group showed no significant difference in the male and female patients.

There was a weak positive correlation between age and CRP (*r* = 0.21, *P* < 0.001).

When 25-(OH) D_3_ and CRP variables are taken as categorical variables and analyzing the relationships between variables, no significant correlation was found (*P* = 1.000).

The prevalence of vitamin D_3_ deficiency in patients with renal failure had a higher number (*P* = 0.000) (Table 6). This frequency were not statistically significant in male patients except advanced-stage renal failure (creatinine < 3.8 mg/dL), (*P* = 0.148).

The frequency of vitamin D deficiency was evaluated between outpatient and hospitalized patients groups in this study population. The incidence of vitamin D_3_ deficiency in all-cause hospitalized patients was more frequent (*P* = 0.000). The prevalence of vitamin D_3_ deficiency in outpatients was 46.3% (*n* = 236) and in hospitalized patients was 67.5% (*n* = 77). The number of subjects with normal vitamin D_3_ was 53.7% for outpatients (*n* = 274) and for hospitalized patients was 32.4% (*n* = 37), respectively (Table 6). Moreover age, ESR, WBC, and CRP medians had higher levels in hospitalized patients.

Finally, B12 levels were measured in 254 patients. The prevalence of low vitamin B12 (<160 pg/mL) was 68% (*n* = 30), and the prevalence of normal vitamin B12 level (>160 pg/mL) was 32% (*n* = 14) in the group with vitamin D_3_ deficiency, whereas the prevalence of low vitamin B12 was 51.4% (*n* = 108), and the prevalence of normal vitamin B12 (Number 30) was 48.6% (*n* = 102) in the normal vitamin D_3_ group. Vitamin B12 deficiency was more frequently seen in patients with vitamin D_3_ deficiency (*P* = 0.043) (Table 6).

## 4. Discussion

Present study did not reflect the true incidence of vitamin D_3_ deficiency because patients who are thought to lack of vitamin D_3_ were included in this study. A limited number of studies conducted in Turkey have shown that vitamin D_3_ deficiency is a common issue during the fall and winter in individuals, particularly for elderly. The deficiency of vitamin D_3_ is seen in 70–75% of women in our country. Vitamin D_3_ deficiency rates are 80–84% in the Middle East, 60–65% in Asia, 50–55% in Europe, and 50% in Latin America [16–18]. Female constitutes the majority of patients may be due to less exposure to the sun and the higher prevalence of osteoporosis.

There was no significant correlation between age and vitamin D deficiency and that may be due to individual characteristics of the studied population. This relationship is shown in some other studies [11, 19]. But many studies did not mentioned the relationship between age and vitamin D levels.

In present study we did not find a relationship between vitamin D_3_ deficiency and inflammatory markers, such as CRP, ESR, and leukocyte counts. Some other studies measured CRP was found the relationship but in these studies the relatively small number of participants were the limiting factor [20, 21]. In several studies were unknown accompanying diseases, and hospitalization rates [9, 22]. There were no studies evaluating ESR, and leukocyte counts were evaluated in 25-(OH) D_3_ deficiency.

Sensitive CRP that was not measured is the limitation of the study. To resolve this drawback was categorized patients according to the levels of CRP. Therefore we divided our study populations into subsets according to CRP levels. Firstly, we counted the number of patients with and without vitamin D_3_ deficiency in CRP normal group. We found no significant difference between two subgroups. Secondly, we separated the study population into CRP normal and significantly high CRP groups. We found no significant difference between the last subgroups again. The reason for this classification was to evaluate the frequency of vitamin D_3_ deficiency in out-patients with important high level of CRP. Finally, we applied correlation analysis between the level of CRP and 25-(OH) D_3_. But a relationship between the level of CRP and 25-(OH) D_3_ was not found in all our analyses. In other words, we did not observe an association between vitamin D deficiency and CRP levels anyway.

Patients' age, serum albumin, CRP, and ESR levels, leukocyte counts, and creatinine values were significantly different between ambulatory and hospitalized patients. The medians of inflammatory markers of hospitalized patients were higher compared to those of ambulatory patients except albumin levels. In addition, the frequency of 25-(OH) D_3_ deficiency was higher once again in hospitalized patients. These also mean that 25-(OH) D_3_ deficiency aggravates all-cause diseases, which is associated with the course of inflammation and infection but not CRP levels.

The prevalence of vitamin D_3_ deficiency in patients with CKD was more common at all stages in female patients; however, it was more common at advanced stage in male patients. This could be explained by a combination of factors, such as poor nutrition or a lack of skin synthesis due to low sun exposure [23]. In CKD patients, dietary restriction and loss of appetite due to uremia or high levels of fibroblast growth factor 23 may be stronger determining factors for 25-(OH) D_3_ deficiency.

In groups without renal failure, creatinine values of vitamin D_3_ deficient patients were lower than vitamin D_3_ normal subjects. Vitamin D_3_ deficient patients had higher PTH values. Higher PTH values were known and expected to be higher among the vitamin D_3_ deficient patients [23]. However, the low level of creatinine was not been described previously, and this difference was statistically significant.

In our study population, the levels of albumin were lower in vitamin D_3_ deficient patients than in vitamin D_3_ normal participations. However, this state did not reach statistical significance. This also pointed out other studies [24]. It has been reported decreased level of albumin in a large scaled study of Melamed et al. [9].

In groups without renal failure, low creatinine and albumin levels might be associated with a nutritional disorder or other comorbid inflammatory-infectious status. It is known that deficiency of vitamin D_3_ and malnutrition were related to each other. Some studies demonstrated that the replacement of vitamin D_3_ did not correct mortality [19]. Patients with high mortality despite treatment with vitamin D_3_ could have other disorders. To clarify this state we evaluated another vitamin such as vitamin B12. Vitamin B12 deficiency was more common in vitamin D_3_ deficient patients. Multivitamin deficiency was common in malnourished and elderly patients, but there was no study that tested vitamin B12 levels in vitamin D_3_ deficient patient in the literature.

For more accurate assessment it is necessary to know other factors that trigger inflammation and infection in studies examining the relationship between vitamin D and inflammatory markers. For example, when hospitalized patients are included in our analysis; all inflammatory markers gained significance statistically. 

In addition, the reason for the deficiency of 25-(OH) D_3_ should be known in similar studies. However, there may be no relationship in encountered 25-(OH) D_3_ deficiency due to low sunlight exposure, and it could be expected in patients with 25-(OH) D_3_ deficiency due to malnutrition. 

According to the results of our study, high levels of CRP in vitamin D deficient patients might be related to other factors such as infectious, inflammatory status, malnutrition, cachexia, or multivitamin deficiency. These factors and others may affect high morbidity and mortality in patients with vitamin D_3_ deficiency. Therefore, replacement of vitamin D alone could be corrected only in patients with vitamin D deficient patients in the foreground.

## Figures and Tables

**Table 1:** Demographical data.

	*n*
Patients with CKD	340
Gender (M/F)	1897 (377/1520)
Outpatient (%)	1717 (90.5)
Hospitalized patient (all-cause) (%)	180 (9.5)
The elderly group	
65–74 years	320
75–84 years	209
>85 years	13

CKD: chronic kidney disease; M: male; F: female; *n*: the number of participants.

**Table 2:** Clinical and laboratory data.

Variable (*n*)	Mean ± SD (Min–Max)
Age (year) (1897)	55 ± 15 (18–90)
25-(OH) D_3_ (μg/L) (1897)	16 ± 13 (3–201)
Vit B12 (pg/mL) (443)	401.9 (84–2001)
PTH (pg/mL) (1162)	112 ± 184 (2–2500)
CRP (mg/L) (996)	12.9 ± 26.7 (2–219)
ESR (mm/h) (1314)	29 ± 21.7 (1–141)
WBC (10^3^/μg/L) (1451)	7.7 ± 3.5 (2.6–100)
Hemoglobin (gr/dL) (1001)	12.5 ± 1.7 (6.6–17.8)
Platelet (10^3^/μg/L) (1003)	264 ± 82 (9–765)
Albumin (gr/dL) (577)	4.13 ± 0.58 (1.6–5.0)

Vit B12: vitamin B12; PTH: parathormone; CRP: C-reactive protein; ESR: erythrocyte sedimentation rate; WBC: white blood cells; SD: standard deviation; Min: minimum; Max: maximum.

**Table 3:** Vitamin D_3_ deficient and normal group medians in all outpatients participants.

(Total number)	25-(OH) D_3_ < 9.9 *μ*g/L		25-(OH) D_3_ > 21 *μ*g/L		*P*
	*n*	Median (Min–Max)	*n*	Median (Min–Max)	
Age (*n* = 1032)	520	54 (18–88)	512	56 (18–87)	0.139
Albumin (*n* = 292)	163	4.3 (2.4–5)	129	4.4 (3–5)	0.071*
CRP (*n* = 554)	262	3.6 (3–204)	283	3.5 (2–156)	0.943
Creatinine (*n* = 770)	386	0.9 (0.3–10)	384	0.9 (0.4–12)	0.018
PTH (*n* = 655)	335	72.8 (3–1675)	320	57 (4–2269)	0.000
ESR (*n* = 716)	353	23 (2–131)	363	23 (2–109)	0.204*
WBC (*n* = 767)	392	7.2 (3.2–22.4)	375	7.1 (3.1–16.3)	0.780
Vitamin B12 (*n* = 254)	138	301 (84–2001)	116	336 (111–1454)	0.115

CRP: C-reactive protein; ESR: erythrocyte sedimentation rate; WBC: white blood cells; Min: minimum; Max: maximum.*When hospitalized patients included in the analysis; albumin and ESR gained significance (*P* = 0.001, *P* = 0.024, resp.)

**Table:** (a)

	25-(OH) vitamin D_3_ < 9.9 *μ*g/L		25-(OH) vitamin D_3_ > 21 *μ*g/L		*P*
	*n*	Median (Min–max)	*n*	Median (Min–Max)	
Age (*n* = 706)	348	54 (18–85)	358	55 (18–87)	0.373
Albumin (*n* = 227)	125	4.4 (2.4–5)	102	4.4 (3–5)	0.355
CRP (*n* = 401)	186	3.5 (3–158)	215	3.5 (2–105)	0.722
Creatinine (*n* = 706)	348	0.8 (0.3–1.3)	358	0.9 (0.4–1.3)	0.000
PTH (*n* = 426)	216	72.9 (3–1013)	210	58 (16–118)	0.000
ESR (*n* = 543)	260	23 (2–107)	283	23 (2–109)	0.639
WBC (*n* = 605)	299	7.15 (3.2–22.4)	306	7.1 (3.1–16.1)	0.990

CRP: C-reactive protein; PTH: parathormone; ESR: erythrocyte sedimentation rate; WBC: white blood cells; Min: minimum; Max: maximum.

**Table:** (b)

	25-(OH) D_3_ < 9.9 *μ*g/L		25-(OH) D_3_ > 21 *μ*g/L		*P*
	*n*	Median	*n*	Median	
Age (*n* = 178)	114	69 (22–90)	64	62.5 (18–83)	0.017
Albumin (*n* = 109)	81	3.4 (1.6–4.9)	28	4.05 (2.7–4.8)	0.001
CRP (*n* = 102)	61	15 (3–218)	41	6 (3–205)	0.067
Creatinine (*n* = 178)	115	2.1 (1.4–13.6)	63	1.75 (1.4–12.4)	0.289
PTH (*n* = 121)	77	194.5 (6–2166)	44	141 (2–2269)	0.161
ESR (*n* = 106)	63	48 (2–131)	43	33 (8–141)	0.003
WBC (*n* = 144)	97	8.4 (0.1–44.4)	47	7.6 (3.7–21.7)	0.641

CRP: C-reactive protein; PTH: parathormone; ESR: erythrocyte sedimentation rate; WBC: white blood cells; SD: standard deviation; Min: minimum; Max: maximum.

**Table 5:** Vitamin D_3_ levels in patients with inflammation and without inflammation.

	*n*	25-(OH) D_3_ Median (Min–Max)	25-(OH) D_3_ (Mean ± SD)
CRP < 6 mg/dL	605	15 (3–79)	17.42 ± 12.21
CRP > 6 mg/dL	390	15 (3–201)	17.47 ± 15.65

CRP total	995	15 (3–201)	17.44 ± 13.65

CRP: C-reactive protein; SD: standard deviation.

**Table 6:** The frequency of CRP, vitamin B12, hospitalization, and renal failure in 25-(OH) vitamin D_3_ deficiency.

	25-(OH) D_3_ < 9.99 *μ*g/L, *n* (%)	25-(OH) D_3_ > 21 *μ*g/L, *n* (%)	*P*
CRP < 6 mg/dL	180 (84.5)	193 (90.2)	
CRP > 30 mg/dL	33 (15.5)	21 (9.8)	
Total	**213**	**214**	**0.770**
Vit B12 < 160 pg/mL	33 (21.7)	15 (12.4)	
Vit B12 > 160 pg/mL	119 (78.3)	106 (87.6)	
Total	**152**	**211**	**0.043**
Albumin < 3.5 g/dL	46 (21)	11 (7.6)	
Albumin > 3.5 g/dL	173 (79)	133 (92.4)	
Total	**219**	**144**	**0.001**
Outpatients	520 (87.1)	513 (93.3)	
Inpatients	77 (12.9)	37 (6.7)	
Total	**597**	**550**	**0.000**
With CKD	115 (24.8)	63 (15)	
Without CKD	348 (75.2)	358 (85)	
Total	**463**	**421**	**0.000**

CRP: C-reactive protein; CKD: chronic kidney disease.
